# Editorial: Harnessing the potential of FLASH: questions we must address prior to effective clinical translation

**DOI:** 10.3389/fonc.2024.1515325

**Published:** 2024-11-13

**Authors:** Per Rugaard Poulsen, Kevin M. Prise, Ranald I. MacKay

**Affiliations:** ^1^ Danish Centre for Particle Therapy, Aarhus University Hospital, Aarhus, Denmark; ^2^ Patrick G Johnston Centre for Cancer Research, Queen’s University Belfast, Belfast, United Kingdom; ^3^ Christie Medical Physics and Engineering, The Christie NHS Foundation Trust, Manchester, United Kingdom

**Keywords:** FLASH, ultra-high dose rate, radiotherapy, radiation oncology, clinical translation

FLASH is radiotherapy delivered with ultra-high dose rates (UHDR) that are several hundred times higher than those currently used clinically. Pre-clinical FLASH studies have shown reduced normal tissue toxicity with unaltered tumour response compared to conventional dose rates (CONV) (1). It promises great potential for cancer treatment with reduced side effects, and the first clinical trial using FLASH dose rates has demonstrated safe use for palliative treatment of bone metastases (2). However, many questions related to biology, physics and oncology remain open regarding safe clinical translation for curative cancer care. Osteoradionecrosis seen in single-fraction dose escalation of FLASH for spontaneous feline (3) and canine (4) oral tumours is a warning that FLASH must be introduced with caution. A rushed, premature clinical implementation with unforeseen toxicity would be harmful not only for the involved patients but for the entire field of FLASH.

This Research Topic features eight articles aiming to identify and address important knowledge and technology gaps that must be filled for effective clinical translation of FLASH for curative cancer treatments. Three contributions are review papers investigating how existing literature may inform clinical translation. Pogue et al. reviewed work on acute skin toxicity in mice describing how beam parameters and oxygenation state impact the FLASH sparing and how the murine studies may guide FLASH studies on higher level species and eventually clinical translation. Many translational human studies will expectedly focus on skin lesions as a safe first choice for FLASH trials and more studies on late toxicity and pig skin are needed to bridge the gap of FLASH efficacy in humans.

McGarrigle et al. presented a systematic evaluation of 41 preclinical studies of ultra-high dose rate radiotherapy. They used a semi-quantitative approach where normal-tissue preservation and tumour control probabilities were evaluated considering the outcomes of each experiment. They showed a correlation between UHDR and FLASH sparing effects with significant association between a normal tissue sparing score index, a therapeutic score index and pulse dose rate.

Fenwick et al. considered the physics advances and pre-clinical characterization that are required to maximise the potential of proton FLASH. They discussed solutions including the use of 3D ridge filters for Bragg peak delivery, creating multi-field plans in which critical normal tissues are only irradiated with a single field, building dynamic systems for proton range modulation and pull-back that allows online plan adaptation. They recommended to select tumours that are normally treated with stereotactic radiotherapy or have low α/β ratios and to develop planning algorithms that simultaneously optimize dose rates and doses to maximise the UHDR coverage of high-dose structures where serious toxicities normally arise.

Lövgren et al. explored the practicalities of treatment planning to deliver Bragg peak FLASH. A research treatment planning system was used to simulate monoenergetic spot scanned protons traversing through a conformal energy modulator, a range shifter, and an aperture. A dose rate constraint of at least 40Gy/s was included in each FLASH proton plan optimisation, and plans were compared to conventional intensity modulated proton therapy. The paper highlighted both the physical restrictions imposed by the move to Bragg peak FLASH and the potential degradation in conventional plan metrics when optimising for FLASH.

Three papers reported on new original radiobiology studies demonstrating both large normal tissue sparing and unaltered tumour efficacy of FLASH. Kristensen et al. investigated the tissue-sparing effect in mice of pencil beam scanning proton FLASH in the spread-out Bragg peak (SOBP). Through full dose response curves the authors found that FLASH needed 40% higher dose than CONV to induce the same acute skin toxicity risk. For the late effect of fibroses, a smaller but still significant dose modification effect of 18% was found for FLASH. The demonstration of maintained FLASH sparing in the SOBP is important since treatment in the Bragg peak is needed for proton FLASH with uncompromised dose conformality.

Liljedahl et al. examined survival of rats with glioblastoma after single-fraction electron beam FLASH and CONV brain irradiation. Doses of 20 Gy and 25 Gy increased survival similarly for FLASH and CONV compared to unirradiated control animals while 30 Gy did not prolong survival for neither FLASH nor CONV. The study adds to the increasing bulk of experimental evidence of equal anti-tumour efficacy for FLASH and CONV. It is important to rule out the risk of a FLASH sparing effects in tumours before safe clinical translation.

Gjaldbæk et al. investigated the safety of single-fraction high dose electron beam FLASH for superficial non-oral tumours in canine patients. The study found no severe adverse effects for treatments up to 30 Gy whereas 35 Gy resulted in severe adverse effects (ulceration) in four out of seven treated sites. Unlike previous oral tumour studies (3, 4) there was no sign of osteoradionecrosis six months or more post-treatment. For non-oral tumours, 30 Gy seemed to be the maximally tolerated dose for single fraction. Finding the maximal dose that can safely be delivered in a single fraction with FLASH is important for clinical translation.

Konradsson et al. investigated improved safety of electron beam FLASH delivery with a modified clinical accelerator by an upgraded beam control system with three independent beam monitoring methods (rather than only using the preset number of pulses). Furthermore, an interesting method for fine-tuning the dose of the first pulse in a sequence was proposed and implemented. It allows adjustment of the fraction dose such that is not limited to integer values of a fixed pulse dose on the order of 1-2 Gy. The improved safety and dose fine-tuning are both highly relevant for clinical translation.

In summary, the papers in this Research Topics address important questions for effective clinical translation of FLASH such as the dose and dose rates needed to trigger normal tissue FLASH sparing, avoidance of unwanted sparing of tumour, maintenance of high dose conformality in FLASH treatments, boundaries for safe dose escalation, as well as machine limitations and means to overcome these. Although important questions remain, such as potentially lower FLASH sparing for fractionated treatments and for dose-limiting late toxicity, this Research Topic brings important contributions to the efficient clinical translation of FLASH.

## References

[1] Favaudon V Caplier L Monceau V Pouzoulet F Sayarath M Fouillade C. Ultrahigh dose-rate FLASH irradiation increases the differential response between normal and tumor tissue in mice. Sci Trans Med. (2014) 6:245ra93. doi: 10.1126/scitranslmed.3008973 25031268

[2] Mascia AE Daugherty EC Zhang Y Lee E Xiao Z Sertorio M. Proton FLASH radiotherapy for the treatment of symptomatic bone metastases: the FAST-01 nonrandomized trial. JAMA Oncol. (2023) 9:62–9. doi: 10.1001/jamaoncol.2022.5843 PMC9589460 36273324

[3] Rohrer Bley C Wolf F Goncalves Jorge P Grilj V Petridis I Petit B. Dose- and volume-limiting late toxicity of FLASH radiotherapy in cats with squamous cell carcinoma of the nasal planum and in mini pigs. Clin Cancer Res. (2022) 28:3814–23. doi: 10.1158/1078-0432.CCR-22-0262 PMC9433962 35421221

[4] Borresen B Arendt ML Konradsson E Bastholm Jensen K Back SA Munck Af Rosenschold P. Evaluation of single-fraction high dose FLASH radiotherapy in a cohort of canine oral cancer patients. Front Oncol. (2023) 13:1256760. doi: 10.3389/fonc.2023.1256760 37766866 PMC10520273

